# Challenges in Laboratory Diagnosis of the Novel Coronavirus SARS-CoV-2

**DOI:** 10.3390/v12060582

**Published:** 2020-05-26

**Authors:** Nadin Younes, Duaa W. Al-Sadeq, Hadeel AL-Jighefee, Salma Younes, Ola Al-Jamal, Hanin I. Daas, Hadi. M. Yassine, Gheyath K. Nasrallah

**Affiliations:** 1 Biomedical Research Center, Qatar University, P.O. Box 2713 Doha, Qatar; ny1204022@qu.edu.qa (N.Y.); dalsadeq@qu.edu.qa (D.W.A.-S.); hadeel.mohammed@qu.edu.qa (H.A.-J.); ola.l.aljammal@gmail.com (O.A.-J.); hyassine@qu.edu.qa (H.M.Y.); 2 College of Medicine, Member of QU Health, Qatar University, P.O. Box 2713 Doha, Qatar; 3 Department of Biomedical Science, College of Health Sciences, Member of QU Health, Qatar University, P.O. Box 2713 Doha, Qatar; sy1203986@qu.edu.qa; 4 College of Dental Medicine, Member of QU Health, Qatar University, P.O. Box 2713 Doha, Qatar; hdaas@qu.edu.qa

**Keywords:** COVID-19, SARS-CoV-2, viruses, diagnostic challenges, molecular testing, serology

## Abstract

The recent outbreak of the Coronavirus disease 2019 (COVID-19) has quickly spread worldwide since its discovery in Wuhan city, China in December 2019. A comprehensive strategy, including surveillance, diagnostics, research, clinical treatment, and development of vaccines, is urgently needed to win the battle against COVID-19. The past three unprecedented outbreaks of emerging human coronavirus infections at the beginning of the 21st century have highlighted the importance of readily available, accurate, and rapid diagnostic technologies to contain emerging and re-emerging pandemics. Real-time reverse transcriptase-polymerase chain reaction (rRT-PCR) based assays performed on respiratory specimens remain the gold standard for COVID-19 diagnostics. However, point-of-care technologies and serologic immunoassays are rapidly emerging with high sensitivity and specificity as well. Even though excellent techniques are available for the diagnosis of symptomatic patients with COVID-19 in well-equipped laboratories; critical gaps still remain in screening asymptomatic people who are in the incubation phase of the virus, as well as in the accurate determination of live viral shedding during convalescence to inform decisions for ending isolation. This review article aims to discuss the currently available laboratory methods and surveillance technologies available for the detection of COVID-19, their performance characteristics and highlight the gaps in current diagnostic capacity, and finally, propose potential solutions. We also summarize the specifications of the majority of the available commercial kits (PCR, EIA, and POC) for laboratory diagnosis of COVID-19.

## 1. Introduction

Infectious diseases impose a major health threat globally, leading to 15 million deaths annually [1]. Infectious diseases remain the third leading cause of death in the US [2]. Fifty years ago, researchers and scientists believed that the age-old battle of humans against the infectious disease was virtually over, with humankind the winners. However, the repeated outbreaks of the past two decades including coronaviruses, avian influenza, chikungunya, and cholera have shown the foolhardiness of that position. Even though the percentage of mortality related to infectious diseases has declined [3], at least a dozen “new” infectious diseases have been identified and reported, including AIDS, Legionnaire disease, and hantavirus pulmonary syndrome. Additionally, traditional diseases which appeared to be “on their way out” (such as malaria and tuberculosis) are resurging [2] and, most importantly, the latest coronavirus disease pandemic (COVID-19). This novel virus (SARS-CoV-2) recently emerged in Wuhan-China, causing a new public health crisis threatening the world. As of the 18th of May, a total of 4,820,714 infected cases, and more than 316,998 deaths (mortality rate ~ 7.0%), were reported (WorldOmeter, COVID-19) [4]. In the last twenty years, mankind has faced three different coronavirus outbreaks: SARS-CoV-1 in 2003, MERS-CoV in 2012, and SARS-CoV-2 pandemic in 2019. Irrespective of the underlying nature of these three coronavirus outbreaks, the most sensible and reasonable approaches to prevent and mitigate the adverse consequences of viral epidemics (or pandemics) on humankind require the development of effective surveillance programs, incorporated with laboratory preparedness. In the case of serious biohazards, such as viral outbreaks, diagnostic laboratories play an essential role in the rapid and accurate detection and isolation of new microorganisms using the cornerstone in diagnostic virology, which are the molecular diagnostic techniques [5,6]. Additionally, the introduction of rapid molecular diagnostic techniques and rapid serological assays in the reference diagnostic laboratories would enable the rapid identification, isolation, and treatment of COVID-19 positive cases. This demonstrates, once more, that laboratory medicine is integral to most care pathways [7] and will perhaps remain so for many years to come. In this review, we will discuss the currently available molecular tests and serological diagnostic tests (laboratory-based and point of care (POC) technologies) used for COVID-19 diagnosis. In addition, we will summarize the associated vulnerabilities and gaps in the performance of the current diagnostic technologies that are likely to have serious consequences against the global efforts to contain the outbreak.

## 2. The Roles of Diagnostic Testing in the SARS-CoV-2 Pandemic

The primary goal of the epidemic containment of COVID-19 is to reduce the infection transmission in the population by reducing the number of susceptible persons or by reducing the basic reproductive number (R0). The R0 is modulated by several factors, including the duration of viral shedding, the infectiousness of the organism, and the contact matrix between infected and susceptible persons [8]. Due to the lack of effective vaccines or treatments, the only available method to reduce SARS-CoV-2 transmission as much as possible is by identifying and isolating infected patients who are contagious and can transmit the diseases. Unfortunately, the rapid spread of COVID-19 outbreak across the globe has exposed the major gaps and vulnerabilities in the abilities of healthcare systems of most countries to successfully contain the outbreak.

The deployment of COVID-19 diagnostic testing has varied widely across the globe. A few countries in Asia showed the power of investment in pandemic preparedness, flexible isolation systems, and intensive case finding in the epidemic containment. For example, in South Korea, they dramatically hindered the COVID-19 outbreak by establishing an unprecedented national testing effort [9] as they successfully managed to perform more than 300,000 tests in the first 9 weeks after identifying the first case of COVID-19 [9]. Similarly, in Singapore, they implemented different protective measures including a broader case definition, aggressive contact tracing, and strict patient isolation [10]. Most importantly, to identify asymptomatic patients who did not meet the case definition, a Singapore-wide screening program on patients with pneumonia, influenza-like illnesses, severely ill patients in ICU, and deaths with a possible infectious cause was performed [11]. Similar approaches were implemented in Taiwan and Hong Kong [12]. These countries successfully contained the COVID-19 outbreak by rapidly deploying resource-intensive strategies that prioritized aggressive testing and isolation to interrupt transmission [12].

Due to the rapid transmission of SARS-CoV-2, the role of diagnostic testing is dependent on the types of test available, the resources required for testing, and the time to obtain results. In other words, the rapid identification of suspected cases remains a high priority to properly allocate personal protective equipment (PPE) and to prevent nosocomial spread with subsequent community transmission [13,14]. Thus, many diagnostic tests for COVID-19 are available so far, with more gaining emergency approval every day. These tests are largely based on four different techniques, as shown in Table 1.

**Figure 1:**
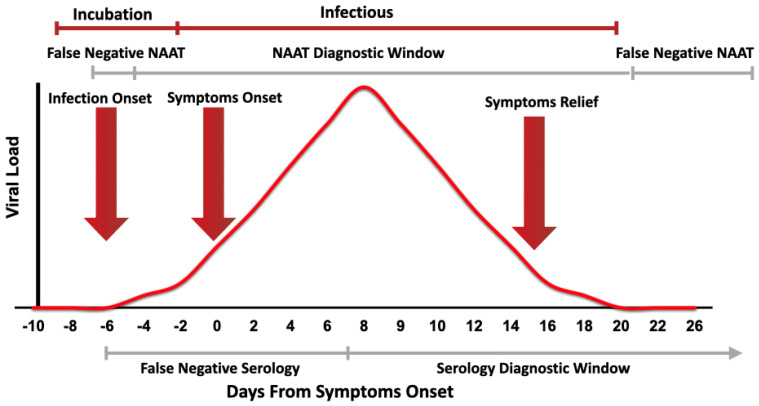
Representative figure showing the correspondence between the viral load during SARS-CoV-2 infection and the clinical course of the disease. The diagnostic windows of nucleic acid amplification tests (NAAT) and serology test are shown. Testing before and after the NAAT diagnostic window will show a false negative result [15]. Nevertheless, testing before the serology diagnosing window will show in false negative results [16].

The current diagnosis of COVID-19 infection relies mainly on the centralized laboratory-based rRT-PCR. Although rRT-PCR provides a relatively rapid result (average 3–4 h), it is limited by transportation to the laboratory and the requirement to batch samples in a large run, as shown in Table 1. Thus, public health sectors are in deep need for fast and reliable tests for SARS-CoV-2 to be able to effectively contain the pandemic. Cost-effective and efficient diagnostic techniques as near to the POC as possible would be a game-changer in the current situation. Some of the currently available POC diagnostic devices utilize molecular-based techniques, and thus are more suitable for diagnosing new COVID-19 cases, while others utilize serological techniques, and thus are better suited to determining whether an individual has previously been infected, to ascertain their suitability to return to frontline services. Figure 1 summarizes the diagnostic window for molecular-based techniques and serological testing.

## 3. Implications and Challenges of Current COVID-19 Diagnostic Tests

### 3.1. Preanalytical and Analytical Errors

Although medical diagnostic errors can happen almost always and everywhere [17], the fragility of diagnostic laboratories is significantly magnified when the healthcare workers were required to face high workload and work in high-throughput settings due to the increasing number of cases [18]. Although the consequences of laboratory errors are often substantial [19], the consequences in the current SARS-CoV-2 pandemic are certainly amplified. Unfortunately, false-positive and false-negative results do not only possess a threat to the health of the individual, but may also disrupt the efficiency of emergency plans, public health policies, and preventive measures applied for containing the pandemic. A false-positive test result not only leads to unrequired treatment but may cause societal problems as it may undermine the workforce available for facing this pandemic if attributed to people working in public facilities. Nevertheless, a false-negative test result may potentially contribute to further spread of the SARS-CoV-2 virus within the community. Therefore, accurate and precise laboratory technologies play a vital role in diagnosing and managing the current SARS-CoV-2 outbreak [20]. However, there are a number of potential preanalytical and analytical errors that must be taken into consideration by clinicians, clinical microbiology laboratories, and public health authorities to avoid false test results.

There is undeniable evidence that the preanalytical phase is the main source of errors in medical laboratories [21,22], accounting for approximately 46% to 68.2% of errors observed during the whole testing process [23], despite continuous improvements in pre-analytical automation. It is estimated that more than one-fourth of all pre-analytical errors result in an unnecessary investigation or inappropriate patient care, substantially magnifying the financial burden on the healthcare system [24], and thus resulting in inadequate and slow healthcare. The safety and quality of diagnostic testing may be endangered by misidentification of the patient and/or sample, collection of an inappropriate or insufficient sample, inaccurate conditions of sample transportation and storage (e.g., prolonged transportation time and injury exposure), presence of interfering substances (e.g., cellular components due to whole blood freezing and inappropriate additives) [25,26,27], and finally, procedural issues occurring during sample preparation, including pipetting errors during manual sample preparation or aliquoting, cross-contamination and sample mismatch [28]. Although analytical errors are believed to be the smallest contributors to laboratory errors, there are several potential analytical problems that could significantly jeopardize the quality of testing, and thus need to be considered. Analytical errors include equipment malfunction, non-adequately validated assays, undetected failure of quality control, active viral recombination, testing carried outside the diagnostic window, poor harmonization of primers or probes, and non-specific rRT-PCR annealing, along with other technical issues [25,26,27].

### 3.2. Chest Computerized Tomography (CT)

Chest computerized tomography (CT) is a conventional, non-invasive imaging technology with high accuracy and speed. The sensitivity to detect SARS-CoV-2 using chest CT is reported to be higher than that of real-time reverse transcription-polymerase chain reaction (rRT-PCR). Recent evidence has shown that asymptomatic patients with COVID-19 may show paradigmatic CT changes very early and even before being positive with rRT-PCR [29,30,31]. For instance, a case was reported in Wuhan city with a history of chills and fever of unknown cause and tested negative four times for SARS-CoV-2 with rRT-PCR from the disease onset [32]. Thus, the clinical physician could not diagnose the patient with COVID-19 at an early stage because of the false negative rRT-PCR results [32]. Therefore, according to Feng et al., patients showing symptoms of fever, dry cough, fatigue, or dyspnea along with recent exposure to SARS-CoV-2 infected patients should be diagnosed with CT despite negative rRT-PCR test results [32]. These pieces of evidence support the advice that the most efficient approach for diagnosing suspected patients with COVID-19 in suspected patients shall encompass a combination of rRT-PCR with clinical and epidemiologic evidence (such as the probability of exposure with infected patient, signs, and symptoms) and chest CT findings.

### 3.3. Nucleic Acid Amplification Testing (NAAT)

Rapid and accurate detection of positive COVID-19 cases is crucial to control the viral outbreak in the community and health care facilities. In general, studies have shown that molecular technologies are more accurate than CT scans and serological tests for the definitive diagnosis of COVID-19, as they can target and identify the specific antigen of SARS-CoV-2. The development of molecular diagnostic technologies against SARS-CoV-2 is dependent upon the understanding of the proteomic and genomic composition of the virus and the viral induction of changes in proteins and genes expression in the patient during and after infection. According to the World Health Organization (WHO), 104 strains of SARS-CoV-2 virus were isolated and sequenced using Illumina and Oxford nanopore sequencing by the 15th of February 2020. By the 24th of March, the genomic and proteomic compositions of SARS-CoV-2 had been identified. However, the host response to the virus is still under investigation. Currently, the NAAT available for SARS-CoV-2 includes rRT-PCR (Laboratory-based) and reverse transcription loop-mediated isothermal amplification (RT-LAMP) (POC) [33,34]. Unfortunately, the currently available diagnostic tests are labor-intensive and time-consuming, and a shortage of commercial kits delays diagnosis.

#### 3.3.1. Manual Laboratory Based NAAT: Real-Time Reverse Transcription-Polymerase Chain Reaction (rRT-PCR)

The current gold standard for the etiological diagnosis of SARS-CoV-2 infection is rRT-PCR on a variety of clinical specimens, including bronchoalveolar lavage fluid, fiber bronchoscope brush biopsies, sputum, nasal swabs, pharyngeal swabs, feces, or blood [35]. The rRT-PCR tests offer several benefits. First of all, rRT-PCR is especially valuable at the early stage of infection, when the viral load is lowest and can differentiate it from other similar viruses, due to its sensitivity and specificity, respectively. Thus, as opposed to serology, rRT-PCR provides more valuable information at the initial stages of infection, as it detects the pathogen directly by detecting its RNA when the aim is to prevent infectivity and antibodies have not yet been built. In addition, rRT-PCR results are generally available within a few hours to 2 days. Moreover, it can be easily operated on a large scale.

Although rRT-PCR offers many benefits, it has some limitations. Its low sensitivity, low stability, and long processing time were detrimental to the health care efforts to contain the outbreak. Also, several external factors may affect rRT-PCR testing results accuracy, including sampling operations, specimen source (e.g., upper or lower respiratory tract), sampling timing (before and after symptoms onset), and the performance of detection kits. Most importantly, recent evidence has shown that the diagnostic accuracy of many of the available commercial rRT-PCR kits for detecting SARS-CoV-2 may be lower than optimal (i.e., <100%), and there are reports where it has given false negatives in subjects for up to two weeks [36,37,38]. This high incidence of false negative diagnosis was observed specifically in SARS-CoV-2 testing. The largest study on coronaviruses testing to date estimated a rate of 41% false negatives on RT PCR diagnostic tests used in China [39,40,41]. However, still more research is needed to determine the true prevalence of such false-negative rRT-PCR results; scientists and researchers agree that the problem is significant, which not only impedes the diagnosis of the disease in patients but also risk patients who assume they are uninfected further transmitting the virus in the community. Moreover, using PCR, codetection with other respiratory viruses is frequently encountered in coronaviruses, and the contribution of positive CoV PCR results to disease severity is not always explicitly exhibited [42]. Furthermore, rRT-PCR requires professionally trained staff to operate sophisticated laboratory facilities, which are usually located at a central laboratory (biosafety level 2 or above), and is often time-consuming, requiring from few hours up to 2 or 3 days to obtain laboratory results. This often leaves a rapidly rising number of potential cases untested and thus opening a gaping hole in SARS-CoV-2 prevention efforts. Furthermore, this time-consuming process of sample testing is not only extremely disadvantageous but also unsafe since the virus needs to be contained. Finally, the US Food and Drug Administration (FDA) concluded that a negative rRT-PCR test result does not completely rule out SARS-CoV-2 infection and shall not be used as a single element for patient management decisions, and re-testing shall be considered in consultation with public health authorities [43]. The information summarized in Table A1 (Appendix A) was extracted from the manufacturer package inserts or their websites.

Protocols for rRT-PCR testing developed by several countries and entities, including Germany, Hong Kong, China CDC, Thailand, and Japan, have been posted to the WHO’s website [44], and the protocol for testing in the United States has been posted to CDC’s website [45]. Table 2 is a comparison between the available rRT-PCR protocols.

#### 3.3.2. Rapid and Point of Care NAAT: Reverse Transcription-Loop-Mediated Isothermal Amplification

Transforming the molecular diagnostic testing for SARS-CoV-2 from laboratory settings to point of care (POC) is potentially important to increase the quantity of testing that can be conducted [39,42], potentially reducing the time to obtain an actionable result, and thus supporting earlier identification of positive cases. Most importantly, POC testing will support the suitable use of quarantine resources, infection control measures, and patient recruitment into clinical trials of treatments. Most of the available molecular POC tests have either gained Conformité Européenne (CE) marking or emergency Food and Drug Administration (FDA) approval [46]. Molecular POC testing utilizes the same basic technology as the laboratory-based assays, but with automating various number of the steps. Therefore, molecular POC tests could be operated in near-patient settings rather than on the laboratory bench, which is expected to reduce the turnaround time and rapidly provides the result. Some of the molecular POC tests utilize isothermal nucleic acid amplification techniques, such as MicrosensDx RapiPrep^©^COVID-19 and Abbott ID NOW COVID-19, while others utilize PCR technology, such as Cepheid Xpert SARS-CoV-2, Credo VitaPC R COVID-19 assay, GenMark ePlex SARS-CoV-2, MesaBioTech Accula SARS-CoV-2, which utilizes lateral flow technology, and the very recent Spartan Cube CYP2C19 System (Canada) [46].

Loop-mediated isothermal amplification (LAMP) was developed as a rapid, accurate, reliable, and cheaper technique to amplify the target sequence at a single reaction temperature instead of sophisticated thermal cycling equipment needed in rRT-PCR [47]. The advantage of using LAMP is that the amount of DNA produced is much higher than in rRT-PCR and a positive test result can be seen visually without requiring a machine to read the results. In addition, it is simple, cheap, and rapid. Several studies evaluated the use of a novel RT-LAMP method against the gold standard rRT-PCR. Two studies showed evidence that RT-LAMP methods demonstrated more than 97% sensitivity targeting the ORF1ab gene compared to rRT-PCR [48,49]. Yang et al. showed that RT-LAMP and rRT-PCR have the same sensitivity and both can detect a 20-fold diluted sample [50]. Additionally, according to Yang et al., the detection limit of LAMP is 1000 copies/mL, which is equal to the rRT-PCR kits [50]. Most importantly, studies have shown that RT-LAMP analysis is extremely specific because it uses six to eight primers to identify eight different regions on the target DNA [50,51]. However, unlike rRT-PCR, LAMP technology does not have such a large background of literature behind it. Thus, tests using LAMP technology for COVID-19 are still being assessed in clinical settings.

Almost all molecular POC described devices are portable benchtop-sized analyzers, except the MesaBioTech Accula and MicrosensDx RapiPrep^©^COVID-19 tests, which are smaller, handheld devices. A variety of clinical sample types may be used, including oral, throat, nasal, or nasopharyngeal swabs. All tests require a similar sample preparation procedure that involves placing the swab sample into the viral transport media and pipetting the sample into a single-use disposable cartridge—this sample preparation step takes approximately 2–10 min [46]. The time to result varies from 13 min in Abbott Diagnostics ID NOW COVID-19 to 45 min in Cepheid Xpert SARS-CoV-2 [46]. The information and validation of each device are summarized in Table A1 (Appendix A).

### 3.4. Serological Testing for COVID-19 Diagnosis

rRT-PCR–based assays performed on respiratory specimens remain the gold standard for COVID-19 diagnostics, as mentioned previously. However, point-of-care technologies and serologic immunoassays are rapidly emerging, with high serological tests for SARS-CoV-2 being at increased demand for better quantification of SARS-CoV-2-positive cases, including asymptomatic and recovered cases. Serological tests are blood-based tests that measure antibodies or antigens present in the blood when the body is responding to a particular infection. Thus, it could identify previous exposure to a particular pathogen as well as the production of the body’s immune system-specific antibodies. Two types of serology test, in particular, are becoming more widely available, namely laboratory-based enzyme immunoassays (EIA) on high throughput automated platforms and rapid, point of care (POC) tests, which are similar to a blood glucose test or home pregnancy test.

**Figure 2:**
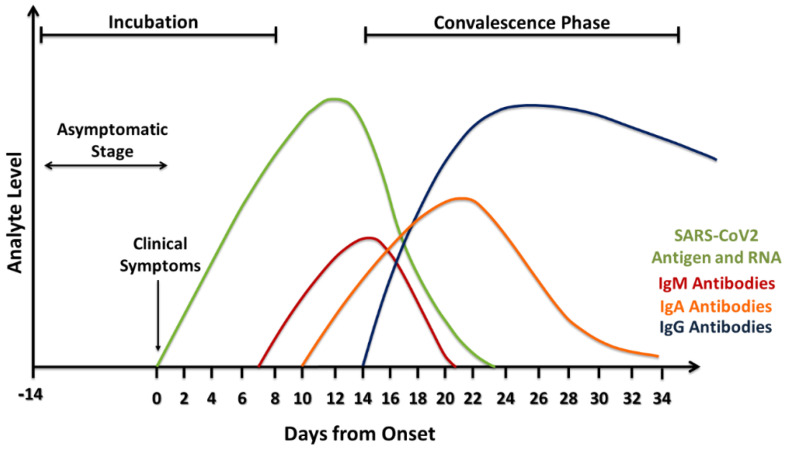
Estimation of biomarker levels during the COVID-19/SARS-CoV-2 infection.

Serological tests offer a number of advantages compared to rRT-PCR. First of all, serological testing can provide further details by identifying individuals who have developed virus-specific antibodies, and thus can detect past infection and give better information regarding the disease prevalence in a population. Unlike viral RNA, virus-specific antibodies stay in the blood for several weeks to months after symptom onset. According to the FDA, IgM antibodies to SARS-CoV-2 are detectable in the blood just a few days after initial infection [52]. However, IgM levels throughout the course of infection are not well characterized. IgG becomes detectable three days from symptom onset or at least 7–10 days after infection [16]. It worth mentioning that when the result is negative for COVID-19, the patient was probably not infected at the time of sample collection. However, that does not mean that he will not get sick. In addition, the detection of SARS-CoV-2 antibodies does not guarantee the protection against COVID-19 infection, as many types of anti-SARS-CoV-2 are not neutralizing antibodies [53]. Considering the fact that 20%–80% of SARS-CoV-2-positive cases are estimated to be asymptomatic, serological tests are especially beneficial because of their scalability, which allows their use on a large scale to assess the overall immune response in a population [54]. In addition, human antibodies are known to be more stable compared to viral RNA, and thus serological samples are less prone to deterioration during sample collection, preparation, transport, storage, and testing compared to rRT-PCR samples. Moreover, serological samples have less variations compared to nasopharyngeal specimens because antibodies are usually homogeneously dispersed in the blood. Furthermore, serological samples can be collected easily with minimal discomfort to the patient during phlebotomy. On the other hand, serological tests have some disadvantages, mainly involving the slow antibody response to SARS-CoV-2 virus, as they may not be detectable until three days from symptom onset or at least 7–10 days after infection (Figure 2) [16]. In addition, these tests are not designed to detect individuals in the early stages of COVID-19 infection. For instance, less than 40% of infected individuals are seropositive (IgM/IgA) in the first seven days, making it unreliable for the detection of acutely infected individuals. Importantly, there have been reports that those with mild cases of COVID-19 infection do not produce antibodies. It was proposed that their innate immune system (cell-mediated immunity) wiped out the virus before the adaptive immune system (antibodies) had to produce antibodies [55]. Since serological tests alone may not be enough to diagnose SARS-CoV-2, combining both serological and molecular techniques would give a valuable diagnostic result.

#### 3.4.1. Manual ELISA

A variety of CE-marked manual enzyme-linked immunosorbent assays (ELISA) have been developed for the rapid detection of neutralizing antibodies (IgM, IgG, and IgA) against the novel coronavirus by many IVD companies such as Euroimmun, Epitope Diagnostics, DRG Diagnostics GmbH, IBL International, Creative Diagnostics and others (Table A2, Appendix A). There are also some commercially available manual ELISA kits for detecting SARS-CoV-2 viral antigens (SP and NP); however, these are mainly used in research and not for clinical diagnosis [56]. Manual ELISA provides accurate and valuable information regarding the immune response to the virus; however, unlike rRT-PCR, it cannot be used for screening or diagnosis of early infection, since specific IgM and IgG antibodies are not detectable at this phase. IgM antibody response occurs earlier than that of IgG, with positive IgM antibodies in 70% of symptomatic patients after 8–14 days and about 90% of total antibodies test positive within 11–24 days [57]. On the other hand, IgG antibodies can be detected around 20 days after viral infection and they persist for a long time [58]. The reactivity of IgG is assumed to reach more than 98% after several weeks, but the extent of this antibody response is yet to be determined [59]. According to recent reports of the WHO, only 2% or 3% of infected COVID-19 individuals appear to have antibodies in their blood. “There is simply not enough data yet to determine if protective immunity is achieved after infection,” says Jennifer Rychert, the medical director of microbial immunology at ARUP Laboratories.

Another challenge of using manual ELISA for SARS-CoV-2 detection is that IgM antibodies are notoriously non-specific, and given the time it takes for the development of specific IgG antibodies, serology testing will not likely play an active role in the detection of early cases (Figure 1) except for diagnosis/confirming late cases or to determine the immunity of healthcare personnel as the outbreak progresses [60]. Furthermore, manual ELISA kits are subject to many interferences, including a specific binding and cross-reaction with other coronaviruses, such as MERS-CoV, SARS-CoV-1, and endemic coronavirus. This depends on the type of antigen used to coat the plates. For instance, an ELISA method based on bat SARSr-CoV Rp3 N protein was successfully developed to detect IgM and IgG antibodies against SARS-CoV-2 in early cases of COVID-19 [59]. A caveat in this ELISA method is that it may produce false positive results since nucleocapsid protein (NP) is the most conserved viral protein among human betacoronaviruses [61]. Hence, antigens used in this ELISA may react with antibodies against other types of coronavirus (HKU1, 229E, OC43, NL63) that are known to cause the common cold [62]. On the other hand, spike protein (SP) is the most diverse protein and several companies have focused on developing ELISA methods for detecting serum antibodies against two domains in the S protein (S1 and S2). The coronavirus envelope spike is responsible for viral entry and it determines the host tropism and virus transmission, which makes it a good candidate for ELISA development [60]. Still, the evaluation of the clinical performance of manual ELISA kits is imperative before using them for COVID-19 diagnosis.

Although many challenges exist, serology testing using ELISA offers great benefits as a therapeutic option to control the current pandemic and possible re-emergence of coronavirus in the future. Hence, the development of manual ELISA kits remains a high priority, as they can complement the existing testing of SARS-CoV-2 by rRT-PCR (the gold standard) and overcome some of its limitations [63].

#### 3.4.2. Automated Serology

The increased demand to perform diagnostic tests on the population imposes a huge clinical and financial burden on diagnostic laboratories. The implementation of automated serological testing has increased the quality assurance and lowered the turn-around-time (TAT) as well as false positive and negative results. Automated techniques are currently adopted for the most commonly used serological methods. Regular serology tests, which are more amenable to automation, are best deployed in the laboratory setting where they can be used to identify immune individuals and for population-level seroprevalence studies. These will be most useful later in the outbreak when the prevalence of the disease increases. In fact, the healthcare market has been flushed with SARS-CoV-2 laboratory testing platforms just a few months into the COVID-19 pandemic. The laboratory-based EIA automated platforms offer high efficiency, high throughput, and improved quality of the results. However, this expansion of newly developed platforms makes it challenging to critically evaluate SARS-CoV-2 laboratory automated tests. Most of the available SARS-CoV-2 manual ELISA kits use the standard 96-microplate as a solid phase and also the standard spectrophotometry/colorimetric method for signal detection, while in the automated EIA assay, the solid phase materials are different, such as polystyrene (PS-COOH) or metal-based nanoparticles (magnetic nanobeads). Further, more sensitive detection systems such as chemiluminescence technology are usually sued in the automated assays.

In April 2020, a fully automated serology test was launched by DiaSorin (Saluggia, **Italy**) to detect SARS-CoV-2 antibodies [64]. The test was developed to detect SARS-CoV-2 IgG antibodies against both the S1 and S2 domain of the spike protein. This increases the specificity of the test and prevents cross-reaction and false-positive results due to other coronaviruses. The LIAISON^®^ XL platform is a chemiluminescence analyzer that is used to perform a fully automated diagnostic tests process with a minimum level of laboratory personnel intervention. The system could perform up to 170 samples per hour to fulfill the need for large population screening for SARS-CoV-2 and identify infected individuals. By the end of April, the DiaSorin test obtained the FDA emergency use authorization (EUA). Similarly, Bio-Rad (Hercules, CA, USA) has developed a test that is blood-based EIA to detect antibodies against SARS-CoV-2 virus [2]. The test could be used manually or on an automated immunoassay platform, including its EVOLIS System. Most importantly, Bio-Rad is working on launching the test globally. In addition, Dynex Technologies, Inc. (Virginia, USA) recently announced that its automated ELISA open platforms are being developed to meet the increased SARS-CoV-2 testing demand by ELISA manufacturers, distributors, and clinical laboratories. Dynex is offering its open platforms and services for the implementation and automation of novel COVID-19 ELISA tests. The company’s core product portfolio consists of microplate ELISA instruments that include 2-plate (DS2^®^), 4-plate (DSX^®^), and 12-plate (high throughput AGILITY^®^) automated ELISA processing systems [65]. Due to the software’s programming capabilities and the quick integration of different tests into Dynex’s open platform, clinical laboratories will be able to rapidly validate and test different COVID-19 ELISA assays and choose the assay that works best for them. Moreover, Eurobio Scientific (Paris, France), a leading company in the field of in vitro medical diagnostics, has launched a new COVID-19 automated serology test developed by its partner Snibe Co., Ltd (Shenzhen, China). Their MagLumi equipment represents an important part of the epidemic’s next phase for precisely defining the population’s immunity to the virus. The machine can process up to 280 samples per hour which makes it very convenient for mass screening. It is very sensitive and robust as it is based on chemiluminescence technology (CLIA) and can be used to perform several serological tests with varying degrees of complexity [66].

#### 3.4.3. Rapid Serological Tests

The development of various serological tests has been permitted to expedite their availability regardless of obtaining EUA from the FDA. However, all antibody tests need to be validated to ensure reliability, accuracy, consistency, and reproducibility [67]. Rapid antibody tests are being explored for testing asymptomatic people who are at the end of their health quarantine period. The test is small, portable, and based on qualitative measurements with either negative or positive results.

Some of the currently available serological POC tests utilize lateral flow immunoassays (Surescreen Diagnostics COVID-19 IgG/IgM rapid test cassette and BioMedomics rapid IgM-IgG combined antibody test for COVID-19). Others utilize time-resolved fluorescence immunoassays (Goldsite Diagnostics Inc. SARS-CoV-2 IgG/IgM Kit), while some are based on colloidal gold immunoassays (Assay Genie COVID-19 rapid POC kit and VivaDiag™ SARS-CoV-2 IgM/IgG rapid test). A summary of the currently available POC devices is presented in Table A3 in Appendix A. All of the described serological POC tests can detect the presence of antibodies from whole blood, plasma, or serum. Generally, they all involve the same basic procedure of pipetting blood from a fingerpick or vein onto the assay, followed by adding the specified buffer solution. Then, the result is displayed within approximately 10–15 min as lines on a display screen. The reference standard used for comparison of the described serological POC tests was rRT-PCR testing. Limited diagnostic accuracy data were collected from clinical, rather than laboratory testing. The largest such study conducted was the evaluation of the BioMedomics IgM-IgG rapid test, which estimated sensitivity of 89% and specificity of 91% among 525 patient samples [54]. Moreover, there is a registered clinical trial protocol for VivaDiag, which anticipates that further clinical accuracy data will become available as the SARS-CoV-2 pandemic proceeds.

### 3.5. Tissue Culture and Neutralizing Test with Actual and Pseudo Virus

Virus neutralization assay (VNA) is a very sensitive and specific method typically used to investigate the antibody response to a virus and study the inhibition of viral replication. This assay is a specialized type of immunoassays because it only detects antibodies that can inhibit virus replication and not all antigen–antibody reactions. This is very important because common antigens may be shared by related groups of viruses, but only some of these antigens are targeted by neutralizing antibodies [68]. VNA can be used for serotyping because a virus serotype is usually based on its neutralization as in poliovirus, which is known to have three major serotypes (neutralization serotypes). Therefore, a successful vaccine against poliovirus must induce neutralizing antibodies to all serotypes (type 1, 2, and 3) to protect from infection [68].

The conventional method of this assay is based on virus inhibition by neutralizing antibodies in cell culture. The titer of neutralizing antibodies can be determined based on the presence/absence of cytopathic effect (CPE) or intracellular staining if using an immunocytochemistry (ICC) technique; and therefore, the highest serum dilution that inhibits infectivity establishes the titer [69]. VNA tests are conducted in four steps including serum dilution, serum and virus incubation, cell culture inoculation, and detection. Although VNA is very sensitive, it is more complex, time-consuming, and requires labor with good technical skills to conduct the assay compared to other serological tests. Currently, VNA tests are done using microtiter plates which are relatively inexpensive and easy to perform using standard laboratory equipment [70].

In the face of the novel COVID-19 epidemic, the development of prophylactic and therapeutic measures has been moving at an accelerated pace by employing a variety of approaches including inactivated whole-virus vaccine, subunit vaccine, viral vector vaccine, and monoclonal neutralizing antibodies. However, due to the significant infectivity and pathogenicity of this virus, biosafety level 3 (BSL3) must be used for handling, which restricts the development of candidate vaccines and therapeutic agents [71]. Pseudovirus, on the other hand, offers several advantages over live virus-based serological assays. While it requires a tissue culture facility, it does not entail high containment measures and can be safely handled in biosafety level 2 (BSL2) cabinets [72]. Therefore, to avoid dealing with infectious viruses, pseudovirus-based neutralization assays (PBNA) are more convenient and feasible for emerging and re-emerging viruses, including MERS-CoV [73], Ebola [74], rabies [75] and the recent novel SARS-CoV-2 [71].

VNA is highly specific and considered to be the gold standard for measuring specific neutralizing antibodies against many viruses in sera samples. The potency of this assay has been previously demonstrated in several studies for confirmatory testing of MERS-CoV [76,77,78]. Observations from these studies showed that neutralization assay was able to detect significant false positive results produced by other serological tests including ELISA. One study reported that all positive IgG ELISA blood donor samples that were retested with PBNA were shown to be negative, indicating cross-reactivity with other circulating human coronaviruses [76]. Also, the study showed that the integration of VNA with the serological testing of MERS-CoV was able to identify even the subclinical infections which highlight the importance of using this assay as a reference test for SARS-CoV-2 detection [76]. Moreover, the VNA assay can be used for studying anti-viral measures against SARS-CoV-2 by evaluating the level of serological cross-reactivity between the virus and antibodies from convalescent serum. A recent study by Nie et al. established and validated a pseudo virus-based neutralization assay (PBNA) for SARS-CoV-2 [71]. The results of this study show significant neutralization potency by antibodies from SARS-CoV-2 convalescent sera. This underlines the future potentials of PBNA in studying and differentiating neutralizing antibodies that are mainly targeting different part of the spike protein [more specifically the receptor binding domain (RBD)] from total antibodies (binding antibodies) that are targeting other viral proteins such the nucleocapsid and membrane proteins. Hence, the outcomes of this assay may aid in finding potential drug targets, and in turn the development of vaccines and antiviral agents. Additionally, it may aid in studying the clinical characteristics associated with the level of neutralizing antibodies in recovered patients.

## 4. Approaches to Improve the Diagnostic Accuracy for COVID-19 Detection

Due to the high infectious rate of SARS-CoV-2, it is essential to have accurate and precise diagnostic technologies as soon as possible, as false-negative test results have shown to have a deleterious epidemiological effect against the global efforts to contain the outbreak [32]. Reducing the number of false-negative test results is vital for determining quarantine measures and cohorts for hospitalized patients. Unfortunately, with so many asymptomatic carriers with false-negative test results, it is very possible that some patients admitted to hospitals for other conditions or trauma may be unknowingly carrying SARS-CoV-2. The healthcare providers need to be able to differentiate between a recovered patient who has cleared SARS-CoV-2 and has antibodies to it and patients who are silent carriers of SARS-CoV-2. This would allow hospitals to prioritize whom to isolate and help immensely to decrease hospital-based transmissions. The following actions can be taken to increase the diagnostic efficacy of the currently available diagnostic techniques. (1) Selecting the optimal sources for specimens when conducting NAAT. Initial investigations showed that the throat and nasal cavity are the most accurate swab sites [32,79] (studies differ on which one is the most accurate). However, the CDC recommends a nasal swab for COVID-19 diagnostic testing using NAAT [80]. (2) Conducting a multi-prong approach (using multiple diagnostic techniques) to confirm the results and reduce the rate of false-negative test results. The establishment of this combined diagnostic workflow of serological testing and NAAT would help in achieving a high-quality, multidimensional, and cost-effective diagnostic efficiency that could meet the detection needs for differential diagnosis, epidemiological investigations, and containing the outbreak of SARS-CoV-2. (3) The multi-prong approach should include diagnostic testing throughout the course of the disease at different time points, ideally from the admission of the patient to the hospital and at a weekly interval [81].

## 5. COVID Diagnostics Technologies/Techniques under Development

### 5.1. Clustered Regularly Interspaced Short Palindromic Repeats (CRISPR-Cas)

CRISPR/Cas-based nucleic acid detection technology was developed with the advantages of sensitivity, specificity, rapidity, and simplicity compared to PCR-based technologies [82,83]. Wang et al. developed an assay that can detect as few as 10 copies of the SARS-CoV-2 in 45 min without a special instrument and showed good consistency with the qPCR assay. Thus, it provides a reliable and straightforward on-site diagnostic method suitable for a local hospital or community testing [83]. Wang et al. successfully developed Cas12a protein, SARS-CoV-2 specific CRISPR RNAs, and a single-stranded DNA (ssDNA) reporter. Furthermore, to enable on-site diagnosis, they labeled the ssDNA reporter with a quenched green fluorescent molecule, which will be cleaved by Cas12a in the presence of SARS-CoV-2 nucleic acid in the detection system, and the resulting green fluorescence can be seen with the naked eye under 485 nm light [83].

### 5.2. Gold Nanoparticles

Gold nanoparticles have been widely reported to guide an impressive resurgence in biomedical and diagnostic applications [84]. The advantages of gold nanoparticle technologies are being simple, rapid, and sensitive, and they facilitate quantitative detection with excellent multiplexing capabilities. Gold nanoparticles were greatly envisioned as state-of-the-art technologies for rapid viral detection [85]. However, to date, there are no available studies regarding the applications of gold nanoparticles for COVID-19 detection. Only one test kit available is based on gold nanoparticle immunochromatography and has attained the CE mark, which is the COVID-19 Colloidal Gold Method Antibody Test from The World Nano Foundation. Although the test still needs to be tested on intact viral RNA from patient samples, it could help relieve the current pressure on PCR-based tests.

### 5.3. Magnetic Resonance Imaging (MRI)

Reports of magnetic resonance imaging (MRI) on diagnosing COVID-19 cases are lacking. Only one study was found to describe the MRI of a patient infected with COVID-19 [86]. The MRI of a patient infected with COVID-19 demonstrated bilateral multilobar focal lung infiltrations, several of which were inhomogeneous with peripheral preference, and some demonstrated direct contact to the visceral pleura, sparing the subpleural space [86]. Nevertheless, according to the American College of Radiology guidelines, practitioners should not perform MRI scans on patients who test positive for COVID-19 or those who are suspected of being infected.

### 5.4. Surface-Enhanced Raman Scattering (SERS)

Surface-enhanced Raman scattering (SERS) spectroscopy has emerged as a powerful analytical technique for molecular analysis (DNA sequences and viral antigens detection), which can be particularly advantageous for diagnostic purposes when combined with inherent optical and chemical properties of plasmonic nanoparticles [87,88]. SERS challenges current fluorescent-based detection methods in terms of both sensitivity and, more importantly, the detection of multiple components in a mixture, which is becoming increasingly more desirable for clinical diagnostics [87,89]. In addition, it can be miniaturized for point-of-care (POC) applications [88,90,91]. However, there are still no available studies of the applications of SERS for detecting SARS-CoV-2.

## 6. Conclusions

Containment efforts of the pandemic will require timely diagnosis, isolation of the infected people to prevent transmission along with extensive community and hospital-based surveillance. The SARS-CoV-2 pandemic has dramatically highlighted the critical role of the diagnostic technologies in the control of infectious diseases. The availability of established diagnostic technologies, which took decades to develop and optimize, has enabled scientists to plug-and-play in the design of SARS-CoV-2 diagnostics [92]. The rapid identification and sequencing of SARS-CoV-2 have enabled the rapid development of NAAT, in which they provided the first line of defense against the ongoing pandemic. After that, serological assays were established because they are easier to administer and to complement NAAT for diagnosing COVID-19 infection. There is now a call for the development of POC and multiplex assays to be rapidly implemented due to the urgent clinical and public health needs to drive an unprecedented global effort to increase SARS-CoV-2 testing capacity. Finally, the blinding speed with which SARS-CoV-2 has spread illustrates the need for preparedness and long-term investments in diagnostic testing.

## Figures and Tables

**Table 1:** Four main type of technologies used for identification of SARS-CoV-2.

Technology	Molecular Tested	Laboratory or Point of Care	Time to Results	Typical Sample Site	Number of Samples/Batches
rRT-PCR	Viral RNA	Laboratory-based	3–4 h	Nasopharyngeal swab, sputum	Up to 96 samples
LAMP	Viral RNA	POC	2–3 h	Nasopharyngeal swab, sputum	1–4 samples
Lateral Flow	Antibody or Antigen	POC	15–20 min	Blood	1 patient sample
ELISA	Antibody or Antigen	Laboratory-based	1–3 h	Blood	Up to 96 samples

**Table 2:** Summary table of available protocols posted to the WHO’s website.

Institute	Gene Targets	Amplicon Size (bp)	Sensitivity	Specificity	Concentration/Volume of Reagents	Does the Protocol Recommend Specific Kits?
China CDC	ORF1ab gene	NR	NR	NR	NR	NR
	N gene	NR	NR	NR	NR	NR
Institute Pasteur, Paris, France	RdRp: nCoV_IP2 gene	108 bp	95% hit rate for approx. 100 copies of RNA GE. LOD for 1x10^7^ RNA copies is ~21 cycles LOD for 1x10^4^ RNA copies is ~30 cycles	No cross reactivity	Final concentration of 0.4 μM of each primer and 0.2 μM of probe	RNA extraction via NucleoSpin Dx Virus and Invitrogen SuperscriptTM III Platinum^®^
	RdRp: nCoV_IP4 gene	107 bp				
	E gene	125 bp				
US CDC, USA	N1 gene	71 bp	LOD: 1x10^0.5^ RNA copies/μL and 10 RNA copies/ μL for Qiagen EZ1 and Qiagen respectively.	Probe showed high sequence homology with SARS coronavirus and Bat Sars-like coronavirus	20 μM primers, 5 μM probe; 15 μL total volume	For the RT-qPCR TaqPathTM 1- Step RT-qPCR Master Mix. For extraction, they recommend bioMérieux NucliSens^®^ systems, QIAamp^®^ kits, QIAGEN kits, Roche Kits and Invitrogen kits
	N2 gene	67 bp				
	N3 gene (removed from diagnostic panel 3/15/20)	72 bp				
National Institute of Infectious Diseases, Japan	N gene	NR	Average Cq value of specimen was 36.7 and 35.0 for the positive control (500 copies of RNA transcript)	NR	1 μL of 20 xprimer and probe mix in a 20 μL reaction with 5 μL of RNA. F primer at 500 nM, R primer at 700 nM, probe at 200 nM.	RNA extracted using QIAamp viral RNA mini kit (Qiagen). Reverse transcription via Super Script IV Reverse Transcriptase (Thermo). RT-PCR via QuantiTect Probe RT-PCR Kit (Qiagen)
Charité, Germany	RdRp gene	NR	LOD: 3.8 RNA copies/ reaction, 95% hit rate; 95% CI: 2.7-7.6 RNA copies/reaction	No reactivity with other human respiratory viruses	RdRP: F-600 nM/reaction, R-800 nM/rxn, P-100 nM each/ reaction,	RNA extracted using MagNA Pure 96 system (Roche), RT- PCR via Superscript III one step RT-PCR system with Platinum Taq Polymerase (Invitrogen).
	E gene	NR	LOD: 5.2 RNA copies/reaction, at 95% hit rate; CI: 3.7-9.6 RNA copies/reaction		E gene: F-400 nM/r reaction, R-400 nM/ reaction, P-200 nM/ reaction	
HKU, Hong Kong SAR	ORF1b-nsp14 gene	132 bp	NR	No reactivity with respiratory cultured viruses and clinical samples.	10 μM primers, 10 μM probes	QIAamp Viral RNA Mini Kit or equivalent and TaqMan Fast Virus Master mix.
	N gene	110 bp				
National Institute of Health, Thailand	N gene	NR	Positive control detected at less than 38 cycles.	NR	40 μM primers, 10 μM probe	Macherey-Nagel Nucleospin RNA virus and Invitrogen superscriptTM III Platinum One-Step Quantitative

NR: not reported; GE: genome equivalent; LOD: Limit of detection.

## Appendix A

**Table A1:** Specifications of the available polymerase chain reaction (PCR) commercial kits for laboratory diagnosis of COVID-19.

Company	Platform	Test Name	Targeted Genes	LOD	Specificity	Comment	Approval
3D Medicines	ABI 7500 Real-Time PCR System	ANDiS^®^ SARS-CoV-2 RT-qPCR Detection Kit	ORF1ab, N, and E genes	5 copies/reaction	Covers 100% of known COVID19 sequences (NCBI and GISAID) No cross reactivity	Automated Near-POC NAAT or POC NAAT	FDA-EUA CE-IVD
KH Medical Co. Ltd.	BioRad CFX96 deep well	RADI COVID-19 Detection Kit	S and RdRp genes	1–10 copies/reaction for S gene 10–50 copies /reaction for RdRp	100%	Manual lab-based NAAT	CE-IVD
SD Biosensor Inc.	Roche LightCycler 480	STANDARD M nCoV Real-Time Detection Kit	ORF1ab and E genes	1–10 copies/reaction	97% for E gene 99% for ORF1a gene	Manual lab-based NAAT	MFDS FDA-EUA CE-IVD
Tib Molbiol	Roche LightCycler 480	ModularDx Kit SARS-CoV-2 (COVID19) E-gene (Tib Molbiol) + LightCycler Multiplex RNA Virus Master (Roche)	E gene	1–10 copies/reaction	NR	Manual lab-based NAAT	RUO
Abbott Molecular Inc.	Abbott m2000 System	Abbott RealTime SARS-CoV-2 EUA test	RdRp and N genes	100 virus copies/mL	Covers 100% of known COVID19 sequences (NCBI and Genebank) No cross-reactivity	Automated Near-POC NAAT or POC NAAT	FDA-EUA CE-IVD
AITbiotech	AITbiotech abCyclerQ, Bio-Rad CFX96™, Applied Biosystems^®^ 7500 Fast Real-Time PCR System	abTES COVID-19 qPCR I Kit	NR	NR	NR	Automated Near-POC NAAT or POC NAAT	CE-IVD
AniCon Labor GmbH	Duplex Real-Time RT-PCR	Kylt^®^ SARS-CoV-2 Confirmation RT-qPCR	RdRP and S genes	10 copies per μL of RNA	Detects all 92 available full genome sequences of SARS-CoV-2 (NCBI)	NR	CE-IVD
Anlongen	NR	nConV-19 Nucleic Acid qPCR Kit	NR	NR	NR	NR	China FDA FDA-EUA CE-IVD
Appolon Bioteck (DAAN Gene Co. Ltd.	ABI 7500, LightCycler 480, AGS4800	Detection Kit for 2019 Novel Coronavirus (2019-nCoV) RNA (PCR-Fluorescence Probing	ORF1ab and N genes	1–10 copies/reaction	96% No cross reactivity with other pathogens	Manual lab- based NAAT	China FDA FDA-EUA CE-IVD
Bao Ruiyuan Biotech (Beijing) Co., Ltd.	NR	Novel Coronavirus(2019-nCov) Nucleic Acid Detection Kit-Multiple Fluorescence PCR	NR	NR	NR	NR	RUO
BGI Health (HK) Co. Ltd.	Roche LightCycler 480	Real-time Fluorescent RT-PCR kit for detection 2019-nCOV	ORF1ab gene	1–10 copies/reaction (100 viral copies/mL) No cross-reactivity with 54 human respiratory pathogens	99% (GenBank and GISAID)	Manual lab- based NAAT	China FDA FDA-EUA
Bioeksen R&D Technologies	Roche LightCycler^®^ 96, Bio-Rad CFX96 Touch™, Qiagen RotorGene^®^ 5 Plex Real-Time PCR Systems	Bio-Speedy SARS-CoV-2 (2019-nCoV) qPCR Detection Kit	RdRp gene	NR	99%	NR	CE-IVD
BIOMAXIMA S.A.	In open PCR systems	SARS-CoV-2 Real Time PCR LAB-KIT	Orf1ab and N genes	10 RNA copies	99% No cross reactivity	NR	CE-IVD
BIONEER Corporation	Exicycler™ 96, CFX96, ABI7500fast	AccuPower^®^ COVID-19 Real-Time RT-PCR kit	E and RdRp genes	NR	NR	Automated Near-POC NAAT or POC NAAT	CE-IVD
BIOTECON Diagnostics GmbH	LightCycler 480 II, Applied Biosystems 7500 Fast, CFX96	Acu-CoronaTM 2.0/3.0 SARS-CoV-2 Real-time PCR Kits	The 2.0 kit targets the E and RdRp genes The 3.0 kit targets RdRp gene	NR	NR	NR	RUO
BIOTECON Diagnostics GmbH	LightCycler 480 II, Applied Biosystems^®^ 7500 fast, CFX96™ RoboPrep 32, KingFisher FlexTM	Virusproof SL SARS-CoV-2 Real-time PCR Kit	E and RdRp genes	NR	100% inclusivity confirmed by in silico analysis with all registered SARS-CoV-2 sequences GISAID database	NR	RUO
Boditech Inc.	NR	ExAmplar COVID-19 real-time PCR kit	NR	NR	NR	Manual lab- based NAAT	RUO
Canvax Biotech	Canvax™ qMAXSen™ qPCR	qMAXSentm Coronavirus (SARS-CoV-2) RT-qPCR Detection Kit	RdRp gene	NR	NR	Manual lab- based NAAT	WHO EUL
CerTest Biotec, S.L.	Bio-Rad CFX96TM Real-Time PCR Detection System, BD MAX™ System	VIASURE SARS-CoV-2 Real Time PCR Detection Kit	ORF1ab and N genes	≥10 RNA copies per reaction for ORF1ab and N gene	No cross reactivity	Manual lab-based NAAT	CE-IVD
CTK Biotech, Inc.	NR	Aridia COVID-19 Real Time PCR Test	NR	95.1% sensitivity	95.9% specificity	Manual lab- based NAAT	CE-IVD
DiaSorin Molecular, LLC	LIAISON^®^ MDX	Simplexa™ COVID-19 Direct RT-PCR Kit	ORF1ab and S genes	LOD for Nasopharyngeal swab: 500 copies/mL LOD for nasal swab: 242 copies/mL	100% No cross reactivity	NR	CE-IVD
Diatheva SRL	CFX96 Biorad, ABI 7500, QuantStudio 5	COVID-19 PCR DIATHEVA Detection kit	RdRp and E genes	100% sensitivity	100%	NR	CE-IVD
Dynamiker Biotechnology (Tianjin) Co., Ltd.	Roche LightCycler 480, ABI 7500	Novel Coronavirus(2019-nCov) RT-PCR Kit	ORF1ab and N genes	NR	NR	Manual lab-based NAAT	RUO
Edinburgh Genetics Limited	Applied Biosystems^®^ 7500 Real-Time PCR System, Roche^®^ LightCycler 480 II	Edinburgh Genetics COVID-19 Real-Time PCR Testing Kit	ORF1ab and N genes	1.0 × 103 copies/mL	100% No cross reactivity	Manual lab-based NAAT	China FDA FDA-EUA CE-IVD
Elabscience	ABI 7500/7500FAST, Roche LightCycler^®^480, BioRad CFX96, BigFish-BFQP16/48 fluorescence quantitative PCR	Novel Coronavirus (SARS-CoV-2) Nucleic Acid Assay Kit (RT-PCR)	ORF1ab and N genes	1 × 10^3^ copies/mL	100% No cross reactivity	NR	RUO
Eryigit Endustriyel Makina ve Tibbi Cihazlar	BioRad CFX Connect (1855201) qPCR	Senteligo Covid-19 qRT PCR Detection Kit	N1, N2 and RNAseP genes	2.57 × 10^2^ copies/mL Sensitivity:99.3%	100 %	NR	CE-IVD
Gene Biosystems	Applied Biosystem^®^ 7500 Real-Time PCR System, Bio-Rad CFX96, Roche^®^ LightCycler 480 II	Gene Bio COVID-19 Qualitative Real Time PCR Kit Ver. 1.0	NR	0.58 copies/μL	NR	Manual lab-based NAAT	RUO
GenomCan Inc.	Roche^®^ LightCycler 480 II, ABI Prism^®^ 7500, Rotor-Gene^®^ 6000, CFX96™	Fluorescent PCR Probe Detection Kit for SARS-CoV-2	ORF1b and N gene	NR	NR	NR	CE-IVD
Genomictree, Inc.	Applied Biosystem^®^ 7500 Real-Time PCR System	AccuraTect RT-qPCR SARS-CoV-2	N gene	100 copies/ reaction	100% No cross reactivity	Manual lab-based NAAT	CE-IVD
GenScript	NR	2019-nCoV qRT-PCR Detection Assay	ORF1ab, RdRp, N and E genes	NR	NR	Manual lab-based NAAT	RUO
Getein Biotech	NR	Novel Coronavirus (2019-nCoV) Real-time RT-PCR Kit	NR	1000 copies/ml	NR	Manual lab-based NAAT	CE-IVD
InBios International, Inc.	CFX96, 7500 Fast Dx	InBios International Smart Detect SARS-CoV-2 rRT-PCR Kit	E, N and ORF1ab genes	7500 Fast Dx: 1.1×10^3^ GE/mL CFX96: 8.6×10^2^ GE/mL	100% No cross reactivity	NR	FDA-EUA
JN Medsys	Real time PCR instrument with FAM detection channel	ProTect Covid-19 RT-qPCR kit	N1, N2, N3 genes	NR	NR	Manual lab-based NAAT	RUO
KogeneBiotech Co. Ltd.	NR	PowerChekTM 2019-nCoV Real-time PCR Kit	NR	NR	NR	Manual lab-based NAAT	EUAL MFDS CE-IVD
KRISHGEN BioSystems	NR	SARS-CoV-2 (Covid-19) Real-Time PCR Kit (as per CDC Atlanta guidelines)	N1, N2, N3 genes	NR	NR	NR	CE-IVD RUO
Krosgen Biotech	LightCycler 480, LightCycler 96, LightCycler Nano, Rotor Gene Q, Mic Realtime PCR, CFX Connect, CFX96	KrosQuanT SARS-CoV-2 (2019 nCOV) Realtime PCR Kit	N1 and N2 genes	NR	NR	NR	CE-IVD
Liming Bio-Products Co., Ltd.	NR	SrongStep^®^Novel Coronavirus (SARS-CoV-2) Multiplex Real-Time PCR Kit	E, N and ORF1ab genes	NR	NR	Manual lab-based NAAT	CE-IVD
Maccura Biotechnology Co., Ltd.	ABI 7500, HONGSHI SLAN-96P, Roche LightCycler 480II	SARS-CoV-2 Fluorescent PCR	ORF1ab, E and N genes	1000 copies/mL	NR	NR	China FDA FDA-EUA CE-IVD
Medical Innovation Ventures Sdn Bhd.	Bio-Rad (CFX96), LineGene 9600 Series, QuantStudio 5	GenoAmp^®^ Real-Time RT-PCR SARS-CoV-2	RdRp, S and N genes	NR	NR	Manual lab-based NAAT	CE-IVD
Ningbo Health Gene Technologies Co. Ltd.	NR	SARS-CoV-2 Virus Detection Diagnostic Kit (RT- qPCR Method)	ORF1ab, N and S genes	NR	NR	Manual lab-based NAAT	RUO
Norgen Biotek Corp	Qiagen Rotor-Gene Q, BioRad CFX96 TouchTM Real-Time PCR Detection System, ABI 7500	2019-nCoV TaqMan RT-PCR Kit	N1 and N2 genes	NR	NR	Manual lab-based NAAT	RUO
Novacyt/primerdesign	Applied Biosystem^®^ 7500 Real-Time PCR System, Bio-Rad CFX ConnectTM Real-Time PCR Detection System, Roche^®^ LightCycler 480 II	Genesig Real-Time PCR COVID-19	NR	0.58 copies/μL of SARS-CoV-2 viral RNA	NR	Manual lab- based NAAT	CE-IVD FDA-EUA WHO EUL RUO
PathoFinder	LightCycler^®^ 480 (Roche), Rotor-Gene^®^ Q (QIAGEN) CFX96™ (Bio-Rad), Mic qPCR Cycler (Bio Molecular Systems), QuantStudio™ 5	RealAccurate Quadruplex Corona-plus PCR Kit	NR	NR	NR	NR	CE-IVD
PaxGen Bio Co. Ltd.	ABI 7500 Real-Time PCR System, 7500 Fast CFX96, SLAN96S	PaxView COVID-19 real time RT-PCR	ORF1ab and N genes	NR	NR	Manual lab-based NAAT	RUO
PerkinElmer Inc.	NR	PerkinElmer^®^ SARS-CoV-2 Realtime RT-PCR Assay	ORF1ab and N genes	20 copies/mL	NR	Manual lab-based NAAT	CE-IVD
Pishtaz Teb Diagnostics	NR	COVID-19 One-Step COVID-19 RT-PCR Kit	RdRp and N genes	200 copies/mL	NR	Manual lab-based NAAT	Iran FDA Certified CE-IVD
Qingdao Jianma Gene Technology Co., Ltd.	Bio-Rad CFX96, ND260	COVID-19 Nucleic Acid Detection Kit (Rapid PCR Fluorescence Method)	ORF1ab gene	1000 copies/ml	NR	Automated Near-POC NAAT or POC NAAT Fast Nucleic acid amplification: 40 min	RUO CE-IVD
Spectrum for Diagnostic Industries (SDI)	M2000rt (Abbott Diagnostics), Mx 3005PTM QPCR System(Stratagene), VERSANTTM kPCR Molecular System AD (Siemens), ABI Prism^®^ 7500 SDS (Applied Biosystems), LightCycler^®^ 480 Instrument II (Roche), Rotor-GeneTM 3000/6000 (Corbett Research),Rotor-Gene Q 5/6 plex Platform (QIAGEN)	SARS-CoV-2 Qualitative Real Time PCR Kit	RdRp and N genes	NR	No cross reactivity	NR	RUO
Systaaq Diagnostic Prouducts	ABI – QuantStudio / StepOnePlus / 7500 Fast 7500, Roche – LightCycler 480, QIAGEN, Rotor-Gene 6000 / Q, BIORAD, CFX96	2019-Novel Coronavirus (COVID-19) Real Time PCR Kit	NR	10 Copies/mL	NR	NR	CE-IVD
Trivitron Healthcare Pvt. Ltd.	NR	NATSure COVID-19 SinglePlex Real-time PCR Kit	Orf1ab and N genes	1 × 10^3^ copies/mL	NR	NR	CE-IVD
Vircell, S.L.	Any qPCR cycler	SARS-COV-2 Real-time PCR Kit	NR	NR	No cross reactivity with common human respiratory CoV or MERS	Manual lab-based NAAT	CE-IVD
Vitassay Healthcare S.L.	Cobas Z480 (Roche) 7500 Fast Real-Time PCR System (Applied Biosystems) II, StepOneTM Real-Time PCR System (Applied Biosystems) II, CFX96 TM Real-Time PCR Detection System (Bio-Rad), AriaMx Real-Time PCR System (Agilent Technologies), DTlite Real-Time PCR System (DNA-Technology), DTPrime Real Time Detection Thermal Cycler (DNA-Technology), Rotor-Gene^®^ Q (Qiagen)I, SmartCycler^®^ (Cepheid)	Vitassay qPCR SARS-CoV-2	ORF1ab and N genes	10 viral RNA copies	No cross reactivity	NR	CE-IVD
Wells Bio, Inc.	NR	CareGENE™ COVID-19 RT-PCR kit	N and RdRp genes	NR	NR	NR	MFDS CE-IVD
Cepheid (US/Worldwide distribution)	GeneXpert Instrument System platform	Xpert SARS- CoV-2	N2 and E genes	250 copies/mL	100%	RT-PCR POC test (time to result: 45 min-1 h)	FDA-EUA
Credo (Singapore)	NR	NRVitaPCR COVID-19 assay	NR	NR	100%	RT-PCR POC test (time to result: 20 min)	CE-IVD
Microsens Dx (London)	NR	RapiPrep COVID-19	NR	NR	NR	LAMP amplification technology POC test (time to result: 30 min)	FDA-EUA
GenMak Diagnostics (United States)	GenMark ePlex instrument	ePlex SARS- CoV-2	NR	1 × 10^5^ copies/mL	1 × 10^6^ copies/μL No cross reactivity	RT-PCR POC test	FDA-EUA
Mesa Biotech (United States)	N/A	Accula SARS- CoV-2	N gene	200 copies/reaction	100%	RT-PCR + lateral flow POC test (time to result: 30 min)	FDA-EUA
Abbott Diagnostics (Worldwide)	ID NOW Instrument	ID NOW COVID-19	RdRp gene	125 GE/mL	100%	Isothermal nucleic acid amplification POC test (time to result: 13 min)	FDA-EUA
Spartan Bioscience Inc. (Canada)	NR	Spartan Cube COVID-19 System	NR	NR	NR	POC test	Health Canada CE-IVD

NAAT: nucleic acid amplification test; GE: genome equivalent; NR: not reported; LOD: limit of detection; RUO: research use only; FDA-EUA: Emergency Use Authorizations: FDA; CE-IVD: European CE Marking for In Vitro Diagnostic; MFDS: Korea’s Ministry of Food and Drug Safety; WHO EUL: The WHO Emergency Use Listing.

**Table A2:** Specifications of the available serological commercial kits laboratory diagnosis of COVID-19.

Company	Test Name	Catalogue Number	Target	Used Antigen	Specificity/Sensitivity	Approval
EUROIMMUN	The Anti-SARS-CoV-2 ELISAs	IgG: EI 2606-9601 G IgA: EI 2606-9601 A	Detection of antibodies (IgG and IgA) against SARS-CoV-2	The S1 domain of the spike protein is used as the substrate in the ELISA	NR	CE
MyBioSource	Human COVID-19 IgG Antibody ELISA Kit	MBS3809906	Detection of the COVID-19 IgG antibody	N protein coated microtiter plate	NR	NR
MyBioSource	Human COVID-19 IgM Antibody ELISA Kit	MBS3809907	Detection of the COVID-19 IgM antibody	N protein coated microtiter plate	NR	NR
MyBioSource	Human Anti-COVID-19 Nucleocapsid Protein (NP) Antibody ELISA Kit	MBS398007	Detection and qualitative measurement of total antibodies against the nucleocapsid protein (NP) of SARS-CoV-2	Recombinant nucleocapsid protein (NP) of SARS- CoV-2 precoated onto the polystyrene microwell strips	Sensitivity: 93.33% Specificity: 95%	CE
MyBioSource	Human SARS-CoV-2 Spike Protein S1 IgG ELISA Kit	MBS2614310	Semi-quantitative detection of human SARS-CoV-2 Spike Protein S1 IgG	Recombinant SARS-CoV-2 protein S1	Detection range 200 U/mL–3.12 U/mL Sensitivity: the minimum detectable SARS-CoV-2 Spike Protein S1 IgG up to 1.2 U/mL.	Manufactured in an ISO 9001:2015 Certified Laboratory.
Biovendor	Human Anti-COVID-19 Spike Protein S1 Receptor-Binding Domain (S1RBD) IgG ELISA Kit	MBS398005	Qualitative determination of human anti-SARS-CoV-2 spike protein S1 receptor-binding domain (S1RBD) IgG antibodies	Recombinant spike protein S1 receptor-binding domain (S1RBD) of SARS-CoV-2 pre-coated onto the polystyrene microwell strips	NR	CE
Epitope Diagnostics Inc.	EDI™ Novel Coronavirus COVID-19 ELISA Kits	IgG: KT-1032 IgM: KT-1033	Qualitative detection of the COVID-19 IgG and IgM in human serum	Microplate coated with COVID-19 recombinant protein.	LOD: 5IU/mL The diagnostic sensitivity is 100%. The diagnostic specificity is 100%.	ISO 13485:2016 certified company CE and FDA certified.
Eagle Biosciences	Coronavirus COVID-19 IgG ELISA Assay	KT-1032	Qualitative measurement of the COVID-19 IgG antibody in serum samples	Microplate coated with COVID-19 recombinant protein.	LOD: 5IU/mL The diagnostic sensitivity is 100%. The diagnostic specificity is 100%.	CE-IVD
RayBiotech Inc.	RayBio^®^ COVID-19/SARS-COV-2 Nucleocapsid Protein ELISA Kit	ELV-COVID19N	Quantitative measurement of COVID-19 N Protein in serum	Antibody specific for COVID-19 N Protein coated on a 96-well plate	The minimum detectable dose of COVID-19 N Protein was determined to be 0.07 ng/mL.	ISO 13485 Certified
RayBiotech inc.	RayBio^®^ COVID-19 Human IgG ELISA Kit	IE-CoVN-IgG	Semi-quantitative measurement of human IgG antibody	SARS-CoV-2 N protein	NR	NR
Creative Diagnostics	SARS-CoV-2 IgG ELISA Kit	DEIASL019	Qualitative detection of novel coronavirus IgG antibodies in human	SARS-COV-2 whole virus lysate antigen is pre-coated	The diagnostic sensitivity is 100%. The diagnostic specificity is 100%.	NR
Creative Diagnostics	SARS-CoV-2 IgM ELISA Kit	DEIASL020	Capture ELISA to detect SARS-COV-2 IgM antibody in human serum/plasma	The anti-µ chain monoclonal antibody is pre-coated on the microplate wells	The diagnostic sensitivity is 100%. The diagnostic specificity is 100%	NR
Creative Diagnostics	SARS-CoV-2 Antigen ELISA Kit	DEIA2020	Quantitative detection of the recombinant SARS-COV-2 nucleoprotein antigen in human serum	The microplate is pre-coated with an anti-SARS-CoV-2 N protein antibody	The LOD of this kit is 1 ng/mL of SARS-COV-2 nucleoprotein	NR
DRG store	Coronavirus COVID-19 IgM	EIA6147	Qualitative measurement of the SARS-CoV 2 IgM antibody in serum	NR	LOD: 5IU/mL	CE
DRG store	Coronavirus COVID-19 IgG	EIA6146	Qualitative measurement of the human anti-SARS-CoV-2 IgG antibody in serum	NR	LOD: 5IU/mL	CE
PISHTAZ TEB DIAGNOSTICS	SARS-CoV-2 IgG ELISA Kit	PT-SARS-CoV-2.IgG-96	Qualitative Determination of the presence of anti SARSCoV-2 IgG	N (nucleocapsid) antigen of the SARS-CoV-2	Sensitivity 94.1% Specificity 98.3%	NR
PISHTAZ TEB DIAGNOSTICS	SARS-CoV-2 IgM ELISA Kit	PT-SARS-CoV-2.IgM-96	Qualitative Determination of the presence of anti SARSCoV-2 IgM	N (nucleocapsid) antigen of the SARS-CoV-2 virus	Sensitivity 79.4% Specificity 97.3%	NR
Vircell Microbiologists	COVID-19 ELISA IgM + IgA	MA1032	IgM + IgA antibodies against SARS-CoV-2 in human serum/plasma	Inactivated SARS-CoV-2 antigens	NR	CE
Vircell Microbiologists	COVID-19 ELISA IgG	G1032	IgG antibodies against SARS-CoV-2 in human serum/plasma	Inactivated SARS-CoV-2 antigens	NR	CE
Tecan-IBL International GmbH	Coronavirus COVID-19 IgM ELISA	30176470	Qualitative measurement of the COVID-19 IgM antibody in serum	Anti-human IgM specific antibody	Sensitivity: 45% Specificity: 100%	CE-IVD
Tecan-IBL International GmbH	Coronavirus COVID-19 IgG ELISA	30176469	Qualitative measurement of the COVID-19 IgG antibody in serum	COVID-19 recombinant full length nucleocapsid protein	Sensitivity: 100% Specificity: 100%	CE-IVD
Creative Biolabs	SARS-CoV-2 (2019-nCoV) Spike Protein ELISA Kit	VCok-Wyb001	Quantitative measurement of natural and recombinant SARS-CoV-2 spike protein	Capture antibody	NR	CE
Creative Biolabs	SARS-CoV-2 (2019-nCoV) Nucleoprotein Protein ELISA Kit	VCok-Wyb002	Quantitative measurement of natural and recombinant SARS-CoV-2 nucleocapsid	Capture antibody	Sensitivity: 42.5 pg/mL	CE
Creative Biolabs	SARS-CoV-2 (2019-nCoV) Anti-NP IgG ELISA Kit	VCok-Wyb005	IgG antibodies to NP of SARS-CoV-2	SARS-CoV-2 nucleocapsid protein	Sensitivity: 93.33%	CE
Creative Biolabs	SARS-CoV-2 (2019-nCoV) Anti-S1 RBD IgG ELISA Kit	VCok-Wyb012	IgG antibodies to S1 RBD of SARS-CoV-2	S1 RBD of SARS-CoV-2 virus	NR	CE
Creative Biolabs	SARS-CoV-2 (2019-nCoV) S1-RBD IgG/IgM ELISA Detection Kit	VCok-Wyb011	IgG or IgM antibodies to S1 RBD of SARS-CoV-2	Spike S1-RBD of SARS-CoV-2	NR	CE

NR: Not reported; LOD: limit of detection; CE: Conformité Européene; CE-IVD: European CE Marking for In Vitro Diagnostic.

**Table A3:** Specifications of the commercially available rapid serological tests for laboratory diagnosis of COVID-19.

Company	Test Name	Catalogue Number	Target	Detection Principle	Specificity/Sensitivity/Accuracy	Approval
AccuBioTech	Accu-Tell COVID-19 IgG/IgM Rapid Test Cassette	ABT- IDT- B352	Detect IgG and IgM antibodies to the COVID-19 Assist in the diagnosis of 1ry and 2ry infection	Rapid chromatographic immunoassay	IgG: Sensitivity: 100% Specificity: 99.5% IgM: Sensitivity: 91.8% Specificity: 99.2% Accuracy: IgG: 99.6%, IgM: 97.8%	CE-IVD
Advaite	RapCov™ Rapid COVID-19 Test	NR	Detect IgG or IgM antibodies to the COVID-19	Colloidal gold complexes containing recombinant 2019 nCoV nucleocapsid antigens	Sensitivity: 89% Specificity: 100% Accuracy: 94.4%	FDA review EUA in progress
Anhui Deep Blue Medical Technology	COVID-19(SARS-CoV2) Ab Test Kit	NR	Qualitative detection of novel COVID-19 IgG/IgM antibodies	Colloidal gold marked recombinant 2019-nCoV antigen	NR	CE-IVD
Assay Genie (Acro Biotech, Inc.) (Ireland)	Rapid POC kit	NR	IgG/IgM	NR	NR	CE
Avioq Bio-Tech Co., Ltd	Novel Coronavirus (2019-nCov) Antibody IgG/IgM Assay Kit (Colloidal Gold)	NR	Qualitative determination of COVID-19 IgG/IgM antibody	Recombinant antigen of 2019-nCoV labeled by Colloidal gold	NR	NR
Aytu BioScience	COVID-19 IgG/IgM Rapid Test	100598	Assay patient antibodies to 2019-nCoV	A solid phase immunochromatographic assay	IgG: Sensitivity: 87.9% Specificity: 100% IgM: Sensitivity: 97.2% Specificity: 100%	CE FDA-EUA
Beijing Wantai Biological Pharmacy Enterprise	Wantai SARS-CoV-2 Ab Rapid Test	WJ-2750	NR	Lateral flow	NR	NR
Biocan Diagnostics	Biocan Coronavirus (COVID-19) IgG/IgM Antibody Test	B521C	Detects and differentiates between an IgM and IgG COVID-19 virus infection for a primary and past infection.	Recombinant COVID-19 antigen conjugated with colloid gold	NR	CE-IVD
BIOHIT HealthCare (Hefei)	SARS-CoV-2 IgM/IgG antibody test kit (Colloidal Gold Method)	NR	Qualitatively determine IgG/IgM antibodies of 2019-nCoV	NR	NR	CE-IVD
Biolidics Ltd.	2019-nCoV IgG/IgM Antibody Detection Kit (Colloidal Gold)	NR	Qualitative detection of IgG/IgM antibodies against SARS-CoV-2	Colloidal gold	Sensitivity: 91.54% Specificity: 97.02%	Singapore HSA CE-IVD
BioMaxima	2019-nCoV IgG/IgM Rapid Test Cassette	1-360-K025	Detection of IgG and IgM antibodies to 2019-nCoV	Lateral flow chromatographic immunoassay	IgG: Sensitivity: 100% Specificity: 98% IgM: Sensitivity: 85% Specificity: 96%	CE-IVD
BioMedomics	COVID-19 IgM-IgG Dual Antibody Rapid Test	NR	Detects IgM/IgG antibodies	Colloidal gold-labeled recombinant novel coronavirus antigen	Sensitivity: 88.66% Specificity: 90.63%	CE-IVD
Biotest Biotech	COVID-19 IgG/IgM Rapid Test Cassette	INGM- MC42	Qualitative detection of IgG and IgM to COVID-19	Specific antigen conjugated gold colloid particles	IgG: Sensitivity: 100% Specificity: 99.5% Accuracy: 99.6% IgM: Sensitivity: 91.8% Specificity: 99.2% Accuracy: 97.8%	CE-IVD
Biotime Biotechnology	SARS-CoV-2 IgG/IgM Rapid Qualitative Test Kit	NR	Detect COVID-19 IgG and IgM antibody	NR	NR	CE
BIOZEK medical	COVID-19 IgG/IgM Rapid Test Cassette	BNCP-402	Qualitatively detect IgM and IgG antibodies to COVID-19	Lateral Flow	Accuracy >92.9%	CE-IVD
Boson Biotech	2019-nCoV IgG/IgM Combo Test Card	1N38C2	Detection of IgG and IgM antibodies Simultaneously	NR	NR	CE
BTNX	Rapid Response COVID-19 IgG/IgM Test Cassette	COV-13C25	Detection of COVID-19 virus IgG and IgM antibody	NR	NR	CE
Camtech Diagnostics Pte Ltd.	camtech COVID-19 Rapid Test Kit	NR	NR	NR	NR	-
Cellex	Cellex qSARS-CoV-2 IgG/IgM Rapid Test	5515C025, 5515C050, 5515C100	Detection and differentiation of IgM and IgG antibodies to COVID-19	Nucleocapsid protein of SARS-CoV-2	Sensitivity: 93.8% Specificity: 95.6%	FDA-EUA CE approval
ChemBio	DPP^®^ COVID-19 IgM/IgG System	65-9569-0	Detection of IgM and IgG antibodies to COVID-19	Nucleocapsid (N) protein of SARS-CoV-2	Sensitivity and specificity values were not released.	FDA
Chemtron Biotech	2019-nCoV IgM Antibody Diagnostic Kit (Colloidal gold)	B202002018	Qualitative detection of COVID-19′s IgM and IgG antibodies	NR	NR	CE-IVD
Dynamiker	2019 nCOV IgG/IgM Rapid Test	DNK-1419-1	Detection of IgG and IgM to COVID-19	NR	92% accuracy.	NMPA
Edinburgh Genetics	COVID-19 Colloidal Gold Immunoassay Testing Kit, IgG/IgM Combined	EGCV0055	Detects IgG and IgM antibodies simultaneously	Colloidal Gold	Sensitivity:98.4% Specificity:99.3%	CE-IVD
Elabscience	Covid-19 IgG/IgM Antibody Rapid Test Kit	UNCOV-40	Screen suspected patients of have been affected by the COVID-19 Qualitative detection of IgG and IgM antibodies to COVID-19	Colloidal gold-labeled recombinant novel coronavirus (COVID-19) antigen	Sensitivity of 98.511%. Specificity of 88.208%	CE
GenBody	COVID-19 IgM/IgG	NR	Rapid and differential detection of IgM and IgG against COVID-19	NR	Sensitivity: 50% at Day 1~6, 91.7% at after Day 7 Specificity: 97.5% Accuracy: 95.2% at Day 1~6, 96.5% at after Day 7	CE-IVD
Goldsite Diagnostics Inc. (China)	GT-100 SARS- CoV-2 IgG/IgM kit	NR	IgG/IgM	NR	IgG: Sensitivity: 100% specificity: 98% IgM: Sensitivity: 85% Specificity: 96%	CE
Guangdong Hecin-Scientific	NR	NR	Tests for IgM against SARS-CoV-2	NR	NR	NR
Guangzhou Wondfo Biotech Co Ltd.	Wondfo SARS- Cov-2 antibody test	W195	Total antibody test (IgG and IgM)	NR	Sensitivity: 86.43% Specificity: 99.57%	China FDA, CE
Hangzhou Alltest Biotech	2019-nCoV IgG/IgM Rapid Test Cassette	INCP-402	Qualitative detection of IgM and IgG antibodies	Lateral flow	NR	CE-IVD
Hangzhou Clongene Biotech Co Ltd.	COVID-19 IgG/IgM Rapid Test Cassette	ICOV3212 ICOV4212	Qualitative detection of COVID-19 IgG/IgM antibodies	Lateral flow	NR	CE-IVD
Innovita Biological Technology	2019-nCoV Ab Test (Colloidal Gold)	NR	Qualitatively detect IgM and IgG antibodies	Immunochromatography with colloidal gold conjugate Lateral flow	NR	ISO13485 CE-IVD
KRISHGEN BioSystems	GENLISA™ Anti-SARS-Cov-2 (Covid-19) IgG/IgM Rapid Test	KBR011	Qualitative determination of IgG/IgM antibodies to Covid-19 (SARS-CoV-2)	Immunochromatography with colloidal gold conjugate	Sensitivity ≥ 80% Specificity: ≥ 98%	CE-IVD
Labnovation Technologies	COVID-19 (SARS-CoV-2) IgM/IgG Antibody Test Ki	NR	Qualitatively detect IgM and IgG antibodies	NR	IgG: Sensitivity: 92% Specificity: 97% IgM: Sensitivity: 82% Specificity: 94%	CE-IVD
Nal von minden GmbH	NADAL^®^ COVID-19 IgG/IgM Test	243003N-25	Qualitatively detect SARS-CoV-2 antibodies (IgM and IgG) Assist in the diagnosis of 1ry and possible 2ry infections	Lateral flow immunochromatographic assay	IgG: Sensitivity: 98.8% Specificity: 98.7% IgM: Sensitivity: 93.7% Specificity: 99.1%	CE
Nanjing Vazyme Medical technology	2019-nCoV IgG/IgM Detection Kit	C6603C	Simultaneous monitoring of IgM and IgG	Antigen colloidal gold of novel coronavirus (COVID-19)	Sensitivity: 91.54% Specificity: 97.02%	CE-IVD
OZO Life	OZO Diamond SARS-CoV2 (COVID-19) lgG/lgM Test	NR	Qualitative testing of new coronavirus SARS-CoV-2	Latex Method	Accuracy: 99.3%	CE-IVD EU- CIBG WHO
OZO Life	OZO India SARS-CoV-2 lgM/lgG Rapid Test Kit	NR	Qualitative testing of new coronavirus SARS-CoV-2	(Colloidal Gold) Lateral Flow	Accuracy: 98%	USFDA CE-IVD EU - CIBG WHO
Phamatech laboratories and diagnostics	COVID-19 “Coronavirus” IgG/IgM Rapid Test Kit	2278	Detection of IgG and IgM antibodies	Utilizes nucleocapsid protein (N-protein) as the binding antigen	IgG: Sensitivity: 100% Specificity: 98.0% Accuracy: 98.6% IgM: Sensitivity: 85.0% Specificity: 96.0% Accuracy: 92.9%	Registered with the FDA
PRIMA home test	PRIMA COVID-19 IgG/IgM Rapid Test	NR	Qualitative determination of COVID-19′s IgM and IgG antibodies	Lateral flow immunochromatographic assay	NR	CE
Ringbio	Novel coronavirus antibody, COVID-19 IgM/IgG Test Kit	C50001	Aiding tool for the testing of COVID-19	Lateral flow immunoassay	NR	CE-IVD Registered in Germany
SD Biosensor	STANDARD™ Q COVID-19 IgM/IgG Duo Test	09COV12B	Specific detection of IgM and IgG to COVID-19 in humoral fluid.	NR	Sensitivity: 81.8% Specificity: 96.7%	Approved for diagnostic use outside the US RUO in US
SensingSelf	COVID-19 Rapid IgG/IgM Combined Antigen Assay Pre-screening Test Kit	FERCSSO5310	Early Detection and Elimination Simultaneous tests for IgM and IgG antibodies	Colloidal gold-labeled recombinant novel COVID-19 antigen	IgG: Sensitivity: 93% Specificity: 97.5% Accuracy: 96.5% IgM: Sensitivity: 82% Specificity: 96% Accuracy: 92.8%	CE-IVD FDA- EUA
Spring Healthcare Services	COVID-19 IgG/IgM Rapid Test (colloidal gold-based)	NR	Tests for 2 antibodies IgM and IgG simultaneously	NR	Sensitivity > 91% Specificity > 99% Accuracy > 97%	CE
Sugentech	SGTi-flex COVID-19 IgM/IgG	COVT025E	Qualitative detection of COVID-19′s IgM and IgG antibodies	NR	Sensitivity: 91% Specificity: 96.67% Accuracy: 94.4%	US FDA listing No. D383895 CE-IVDKorea MFDS Product-license No. 20-213
Sure Bio-Tech	SARS-CoV-2 IgM/IgG Ab Rapid Test	VC012103	Qualitative detection of the antibody IgM/IgG to novel Coronavirus	NR	IgG: Sensitivity: 92% Specificity: 97% IgM: Sensitivity: 82% Specificity: 94%	CE/ISO13485
SureScreen Diagnostics (England)	COVID-19 Rapid Test Cassette	NR	IgG/IgM	NR	NR	CE
VivaChek Biotech	VivaDiag COVID-19 IgM/IgG Rapid Test	NR	NR	NR	NR	CE
Willi Fox	Willi Fox COVID-19 IgM/IgG rapid test	7771730	Detection of IgM and IgG antibodies to COVID-19 from day 7 to 8	NR	IgG: Sensitivity: 98.8% Specificity: 98.7% IgM: Sensitivity: 93.7% Specificity: 99.1%	CE
Willi Fox	Willi Fox COVID-19 Antigen Test	7771730	Directly detect the COVID-19 virus from the second to third day after the infection	NR	Sensitivity: 95.3% Specificity: 98.0%	CE
Xiamen Wiz Biotech	Diagnostic Kit (Colloidal Gold) for IgG/IgM Antibody to SARS-COV-2	NR	Qualitative detection of IgG and IgM antibodies	Colloidal Gold	NR	CE
Zhejiang Orient Gene Biotech	COVID-19 IgG/IgM Rapid Test Cassette	GCCOV-402a	Qualitative and differential detection of IgG and IgM antibodies	Lateral flow	NR	CE

NR: Not reported; HAS: Health Sciences Authority; CE: Conformité Européene; CE-IVD: European CE Marking for In Vitro Diagnostic; RUO: research use only; NMPA: National Medical Products Administration; MFDS: The Ministry of Food and Drug Safety; EU- CIBG: European Union-Centraal Informatiepunt Beroepen Gezondheidszorg (Dutch: Central Health Professions Center; Ministry of Health, Welfare and Sports; Hague, Netherlands).
